# Molecular Cytogenetic Identification of a Novel Wheat–*Thinopyrum ponticum* 1J^S^ (1B) Substitution Line Resistant to Powdery Mildew and Leaf Rust

**DOI:** 10.3389/fpls.2021.727734

**Published:** 2021-10-01

**Authors:** Mingzhu Li, Yanzhen Wang, Xiaojuan Liu, Xingfeng Li, Honggang Wang, Yinguang Bao

**Affiliations:** ^1^State Key Laboratory of Crop Biology, Shandong Agricultural University, Tai'an, China; ^2^Agronomy College of Shandong Agricultural University, Tai'an, China; ^3^State Key Laboratory of Crop Stress Biology for Arid Areas and College of Agronomy, Northwest A&F University, Yangling, China

**Keywords:** *Thinopyrum ponticum*, substitution line, GISH, FISH, powdery mildew, leaf rust

## Abstract

*Thinopyrum ponticum* (2*n* = 10*x* = 70) is a wild relative of wheat with high tolerance to both biotic and abiotic stresses; it has been wildly used in wheat genetic improvement. A disomic substitution line named SN19647 was derived from a cross between *Triticum aestivum* and the wheat–*Th. ponticum* partial amphiploid SNTE20 (2*n* = 8*x* = 56). It was evaluated for disease resistance and characterized *via* sequential fluorescence *in situ* hybridization (FISH)-genomic *in situ* hybridization (GISH) and molecular markers. The results showed that SN19647 carried resistance to both powdery mildew and leaf rust. It contained 42 chromosomes with a pair of wheat chromosome 1B replaced by a pair of J^S^ chromosomes from *Th. ponticum*. In addition to chromosomal substitution events, structural variation also occurred on wheat chromosomes 2A, 5A, 6B, and 7B. Based on marker analysis, 19 markers specific to the J^S^ chromosome were obtained, of which seventeen markers belonged to homoeologous group one. These results indicated that SN19647 was a 1J^S^ (1B) substitution line. Compared with the known 1J^S^ (1D) substitution line CH10A5, it was found that 17 markers generated different specific bands to *Th. ponticum*, confirming the novelty of the 1J^S^ chromosome in SN19647. Therefore, SN19647, resistant to powdery mildew and leaf rust, was a novel 1J^S^ (1B) substitution line that can be used in wheat genetic improvement.

## Introduction

Wheat (*Triticum aestivum* L., 2*n* = 6*x* = 42, AABBDD) is cultivated in diverse geographical regions, environments, and production systems, occupying approximately 220 million ha worldwide (Singh et al., 2016). With the global population estimated to exceed 9 billion by 2050, a 1.6% annual increase in wheat production has been expected to satisfy the increasing population, with the projection of an enhanced yield from 3 to 5 tons/ha (Tilman et al., 2002; Singh et al., 2016). However, climate change and disease occurrence are threatening wheat productivity (Price et al., 2013; Curtis and Halford, 2014; Lobell, 2019).

Powdery mildew is a devastating foliar disease of wheat especially in regions with a cool, maritime climate. It is caused by the fungus *Blumeria graminis* f. sp. *tritici* (*Bgt*). The epidemic that powdery mildew causes will destroy leaf and sheath tissues and significantly reduces tiller number, grain number and kernel weight (Conner et al., 2003), resulting in yield losses. Leaf rust, caused by the fungus *Puccinia triticina* (*Pt*), is another major wheat disease that frequently occurs (Bolton et al., 2008). It causes significant yield losses by affecting biomass, kernel weight and kernel number per acre (Herrera-Foessel et al., 2006). Although fungicides can be used to control these two kinds of diseases, breeding resistant cultivars are considered a cost-effective and eco-friendly approach (Wang et al., 2005; Liu et al., 2017). This breeding process is based on exploration and utilization of resistance genes.

So far, there are 68 (*Pm1* to *Pm68*) powdery mildew resistance genes and 79 (*Lr1* to *Lr79*) leaf rust resistance genes which have been cataloged in wheat and its relatives (Qureshi et al., 2018; He et al., 2021). However, many of them are overcome by new virulent pathogens. Previous studies showed that resistance genes from wheat-related species generally had a wider resistance spectrum and better durability than those from common wheat (Klymiuk et al., 2018; Wang et al., 2018). Therefore, it has become increasingly important to identify new sources of disease resistance from wild relatives via distant hybridization (Wang et al., 2016; Mo et al., 2017).

*Thinopyrum ponticum* (2*n* = 10*x* = 70, E^e^E^e^E^b^E^b^E^x^E^x^StStStSt or JJJJJJJ^S^J^S^J^S^J^S^), a perennial wild relative of wheat, has proven to be a valuable source for resistance to various wheat diseases (Li and Wang, 2009). Up to now, there have been 11 genes formally designated from *Th. ponticum*, including powdery mildew resistance gene *Pm51* and leaf rust resistance genes *Lr19, Lr24* and *Lr29* (Li and Wang, 2009; Zhan et al., 2014). However, none of them originated from the homoeologous group one of *Th. ponticum*. Recently, the 1J^S^ chromosome of *Th. ponticum* in the 1J^S^ (1D) disomic substitution line CH10A5 was reported to be probably responsible for resistance to both powdery mildew and stripe rust (Wang et al., 2020).

In this study, a novel wheat–*Th. ponticum* 1J^S^ (1B) disomic substitution line with resistance to powdery mildew and leaf rust was developed from a hybrid of the common wheat cultivar Jimai22 and wheat-*Th. ponticum* octoploid SNTE20, and named SN19647. Methods of disease evaluation, genomic *in situ* hybridization (GISH), fluorescence *in situ* hybridization (FISH) and molecular markers were employed to identify its disease resistance, chromosomal composition and variation. The alien chromosomes in SN19647 were also compared with those in CH10A5.

## Materials and Methods

### Plant Materials

The wild relatives used in this study included *Th. ponticum* (2*n* = 10*x* = 70, E^e^E^e^E^b^E^b^E^x^E^x^StStStSt or JJJJJJJ^S^J^S^J^S^J^S^) and *Pseudoroegneria spicata* (2*n* = 2*x* = 14, StSt). *Th. ponticum* (accession No. R431) was provided by Prof. Zhensheng Li, formerly of the Northwest Institute of Botany, the Chinese Academy of Sciences, Yangling, China. *P. spicata* was provided by Prof. Lihui Li, Institute of Crop Science, Chinese Academy of Agricultural Sciences, Beijing, China. Other materials included SNTE20, Yannong15 (YN15), Shannongfu63 (SNF63), Jimai22 (JM22), Huixianhong (HXH), and CH10A5. Wheat-*Th. ponticum* octoploid SNTE20, developed from the cross of *Th. ponticum*/YN15//SNF63, was used as the female parent. A predominant wheat cultivar in Shandong Province which is JM22 was used as the male parent to cross with SNTE20 and backcrossed with the resulting F_1_ progeny, and then SN19647 was selected in the generation BC_1_F_4_. For disease resistance evaluation, HXH served as the susceptible control. The 1J^S^ (1D) substitution line CH10A5, provided by Pro. Changyou Wang, Agronomy College of Northwest A&F University, Yangling, China, was used to compare the 1J^S^ chromosomes in the two substitution lines.

### Evaluation of Powdery Mildew and Leaf Rust Resistance

Seedling powdery mildew resistance was performed in a growth chamber using the *Bgt* isolate E09, following the method stated in the study by Zhao et al. (2013). Seedlings were grown in rectangular plastic trays (5 × 5 cm, 10 plants per tray) and inoculated with fresh *Bgt* conidiospores obtained from the susceptible cultivar HXH at the one-leaf stage. After ~2 weeks, when symptoms were severe on the susceptible HXH, infection types (ITs) on the plants were described using a 0–4 infection scale: 0–2 scores indicate resistance while 3–4 indicate susceptibility. At the adult stage, resistance to powdery mildew and leaf rust was evaluated after natural infection in field-grown plants at the Experimental Station of Shandong Agricultural University over three growing seasons (2018–2020). The most severe reaction type in a given year was considered to be the final resistance results. Fifteen plants were grown in each 1.5-m long row, with 25 cm spacing between the rows. HXH was planted perpendicular and adjacent to the test rows to serve as an inoculum spreader and a susceptible control. The disease symptoms were recorded three times at weekly intervals after flowering, and the most severe infection score was used as the final response. The infection types of powdery mildew and leaf rust at the adult stage were scored using a 0–9 scale (Li et al., 2011) and a 0–4 scale (Roelfs et al., 1992), respectively.

### *In situ* Hybridization

The chromosomes were prepared following the method described in the study by Kato et al. (2004). The purified total genomic DNA extracted from *Th. ponticum* and *Ps. spicata* were labeled with fluorescein-12-dUTP and used as probes, with the sheared genomic DNA from YN15 (AABBDD) as a blocker. Genomic *in situ* hybridization analysis was performed as described in the study by Fu et al. (2012). For FISH analysis, oligonucleotide probes, including TAMRA (6-carboxytetramethylrhodamine)-labeled oligonucleotides pAs1-1, pAs1-3, pAs1-4, pAs1-6, AFA-3 and AFA-4, and FAM (6-carboxyfluorescein)-labeled oligonucleotides pSc119.2-1 and (GAA)_10_, were used. All probes were synthesized by Sangon Biotech Co., Ltd. (Shanghai, China). Fluorescence *in situ* hybridization analysis was performed as described in the study by Huang et al. (2018). The chromosomes were counterstained with 4,6-diamidino-2-phenylindole (DAPI), and the images were captured with a fluorescence microscope (Olympus BX60, Tokyo, Japan) equipped with a charge-coupled device (CCD) camera.

### Molecular Marker Analysis

A total of 914 markers (Table 1), including simple sequence repeat (SSR)/expressed sequence tag (EST), which belonged to the first homologous group (https://wheat.pw.usda.gov/GG3/), intron targeting (IT) markers (Zhang et al., 2017), and PCR-based landmark unique gene (PLUG) markers (Wang et al., 2020), were synthesized by Sangon Biotech Co., Ltd. and used to identify the homeologous group of the alien chromosomes in SN19647 and compare their identity with those in CH10A5. PCR amplification system was a 10-μL reaction mixture, containing 40 ng genomic DNA, 2 μM each of forward and reverse primers, 2.5 mM each of the dNTPs, 2.5 mM MgCl_2_, 1× PCR buffer (10 mM Tris-HCl, pH 8.5, 50 mM KCl), and 0.5 U Taq DNA polymerase. It was carried out on a PTC-200 thermal cycler (Bio-Rad, Hercules, CA). The PCR products were resolved on 8% non-denaturing polyacrylamide gels, and the band patterns were visualized by silver staining.

**Table 1:** Markers used in this study.

**Marker types**	**Numbers**	**References**
SSR/EST	69	https://wheat.pw.usda.gov/GG3/
IT	841	Zhang et al., 2017
PLUG	4	Wang et al., 2020

## Results

### Assessment of Powdery Mildew and Leaf Rust Resistance

**Figure 1:**
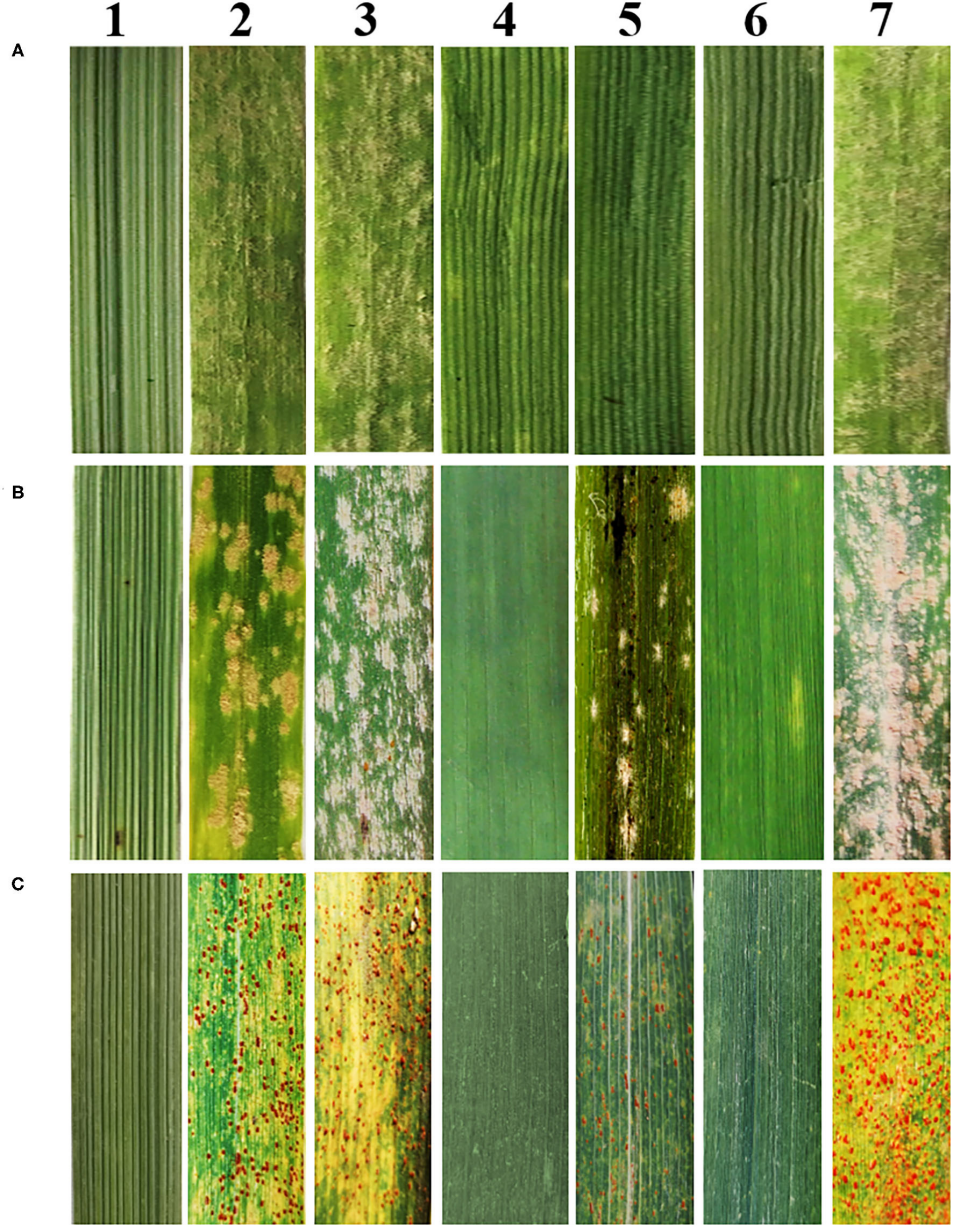
Reactions to powdery mildew and leaf rust. **(A)** Reactions to powdery mildew at the seedling stage. **(B)** Reactions to powdery mildew at the adult stage. **(C)** Reactions to leaf rust at the adult stage. 1–7 refers to *Th. ponticum*, YN15, SNF63, SNTE20, JM22, SN19647 and HXH, respectively.

At the seedling stage, SN19647 and its parents were inoculated with the *Bgt* isolate E09, then the disease reaction was assessed once the susceptible control HXH was thoroughly infected. The results showed that *Th. ponticum*, SNTE20, JM22 and SN19647 were all resistant with an IT score of 0. Whereas the common wheat parents YN15 and SNF63 with an IT score of 4 appeared susceptible. These results suggested that the resistance to powdery mildew at the seeding stage in SN19647 originated from either *Th. ponticum* or JM22 (Figure 1A, Table 2).

**Table 2:** Disease responses of SN19647 and its parents.

**Materials**	**Powdery mildew**		**Leaf rust**
	**Seeding stage**	**Adult stage**	
*Th. ponticum*	0	0	0
YN15	4	7	4
SNF63	4	8	4
SNTE20	0	0	0
JM22	0	6	3
SN19647	0	0;	0
HXHs	4	9	4

At the adult stage, resistance to powdery mildew and leaf rust was tested in the field over three growing seasons (2018–2020), and the most severe reaction type observed in a given year was considered to be the final result. It was found that SN19647, *Th. ponticum*, and SNTE20 were resistant to powdery mildew, while YN15, SNF63 and JM22 showed susceptibility (Figure 1B, Table 2). Similar results were observed for leaf rust resistance at the adult stage in all the examined materials (Figure 1C, Table 2). Thus, these results collectively indicated that the resistance to two types of disease of SN19647 was inherited from *Th. ponticum* at the adult stage.

### Cytological Characterization

**Figure 2:**
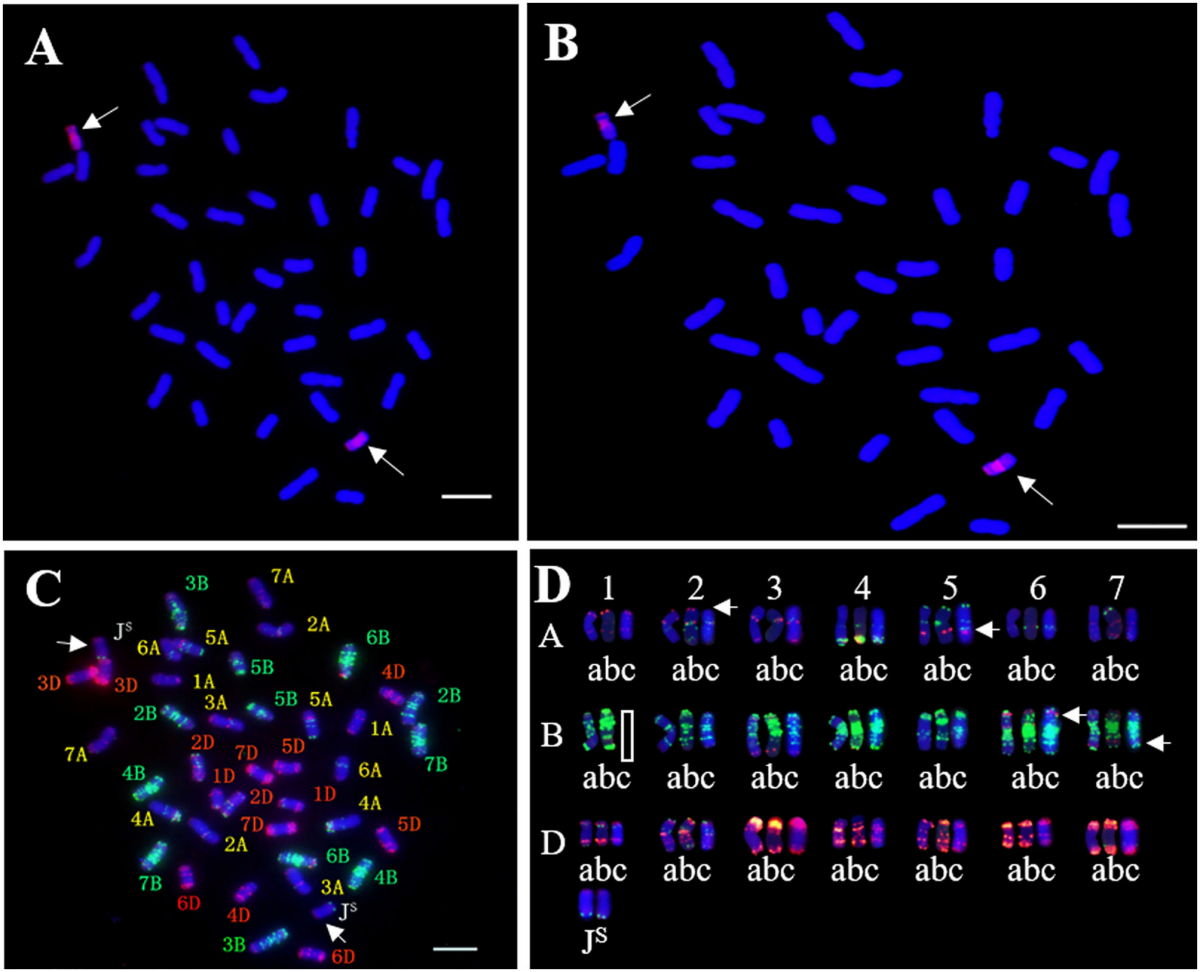
*In situ* hybridization analysis of SN19647. **(A)** GISH pattern of SN19647 probed with *Th. ponticum* genomic DNA. **(B)** GISH pattern of SN19647 probed with *Ps. spicata* genomic DNA. **(C)** FISH pattern of SN19647. **(D)** Comparison of wheat chromosomes in SNTE20 (a), JM22 (b) and SN19647 (c). Arrows indicate the chromosomes J^S^ or locations with different FISH bands. Scale bars = 10 μm.

Genomic *in situ* hybridization and FISH were performed to determine the chromosomal composition of SN19647. When probed with total genomic DNA of *Th. ponticum* and blocked with DNA of YN15, GISH showed that SN19647 contained two *Th. ponticum* chromosomes with distinct and uniform hybridization signals in addition to 40 wheat chromosomes counterstained by DAPI (Figure 2A). When the St-genomic DNA of *Ps. spicata* was used as a probe, the two alien chromosomes showed red signals in the centromeric regions and occasionally in the telomeric regions, which suggested that they belonged to the J^S^ genome (Figure 2B).

After removing the GISH signals, the slide was subjected to FISH analysis using eight probes. The J^S^ chromosome pair produced red and green signals in the terminal regions of the short and long arms, respectively (Figure 2C). The wheat chromosome pair 1B was eliminated and 14 A-(1A-7A), 12 B-(2B-7B), and 14 D-(1D-7D) chromosomes were detected, indicating SN19647 as a disomic substitution line with a *Th. ponticum* J^S^ chromosome pair replacing a wheat 1B chromosome pair. Additionally, FISH patterns of the chromosomes in SN19647 were compared with those in its parents, SNTE20 and JM22. Differences were found in the terminal region of 2AS, the middle of 5AL and 6BS, and the region close to the terminal in 7BL (Figure 2D), which suggested that these chromosomes underwent structural variations with the formation of SN19647.

### Molecular Marker Analysis

**Figure 3:**
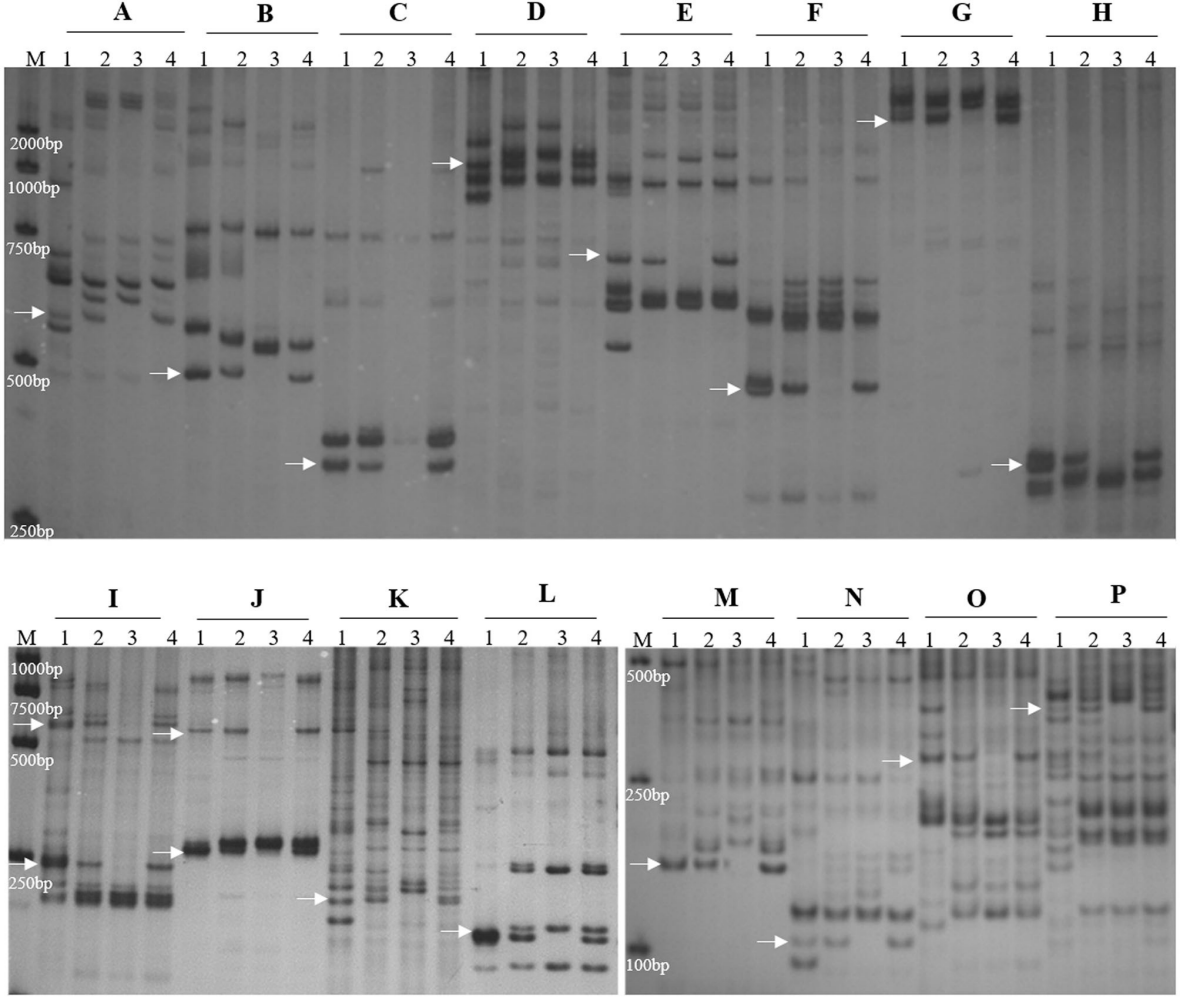
Molecular marker analysis of SN19647. **(A)** CINAU851. **(B)** CINAU857. **(C)** CINAU859. **(D)** CINAU862. **(E)** CINAU865. **(F)** CINAU866. **(G)** CINAU870. **(H)** CINAU880. **(I)** CINAU889. **(J)** CINAU890. **(K)** CINAU971. **(L)** CINAU1616. **(M)** Xgwm268. **(N)** SWES650. **(O)** Xmag3030. **(P)** BE405232. Lanes: M, DL2000 marker; 1-4 refer to *Th. ponticum*, SNTE20, JM22, and SN19647, respectively. Arrows indicate the specific bands of *Th. ponticum*.

To determine the homoeologous group of the J^S^ chromosome introduced to SN19647, 841 IT and 65 EST/SSR primer pairs were screened among *Th. ponticum*, SN19647 and its parents, SNTE20 and JM22. In total, 12 IT markers, 3 EST markers and one SSR marker showed amplification with prominent bands specific to *Th. ponticum* in SN19647, which were absent in the common wheat parent JM22 (Figure 3). Among them, 14 markers, including one SSR, three EST and 10 IT markers belonged to the first homoeologous group (Table 3), indicating 1J^S^ as the introduced *Th. ponticum* chromosome in SN19647. Thus, SN19647 was confirmed as a wheat-*Th. ponticum* 1J^S^ (1B) disomic substitution line.

**Table 3:** Markers specific to the alien chromosomes of SN19647.

**Markers**	**Homeologous groups**	**Sequences**
*CINAU851*	1	F: 5′-TCTTCGTATCAGGCTTTGCTT-3′
		R: 5′-ACCTTGCGGATCCTTCTCAA-3′
*CINAU857*	1	F: 5′-GGGCATGCTCTTTCAACGG-3′
		R: 5′-TCATTTTCCAAACACCCTCAGT-3′
*CINAU859*	1	F: 5′-ATGCAGAGGATGGTCTTCGT-3′
		R: 5′-AGTCGATGAGGCTCTTATTTGT-3′
*CINAU862*	1	F: 5′-AGCCTGTCTCGGTGGTAAAA-3′
		R: 5′-CTGGAGTAGACAGTGCGTTTG-3′
*CINAU865*	1	F: 5′-TTCCGAATGCCTGCTTGTTT-3′
		R: 5′-ACACACATGGAACTCTTCATGA-3′
*CINAU866*	1	F: 5′-TCCAATCATTGCGCCAAATCT-3′
		R: 5′-GCAGTGTCCAAAAGTCCCTT-3′
*CINAU870*	1	F: 5′-TGTTCCGTGGAGATATCTTCCA-3′
		R: 5′-GGAAGGCCATCTGAATCAATTAC-3′
*CINAU880*	1	F: 5′-TGGCAATGTCTGTAGCCATC-3′
		R: 5′-GAACTCAAGCCGTGTCATGG-3′
*CINAU889*	1	F: 5′-TGTTCCAGTTCGAGCAAGAC-3′
		R: 5′-AGGGATCAAGTGGAGAGTGA-3′
*CINAU890*	1	F: 5′-GTTCACGTGCTGCTGAGTAT-3′
		R: 5′-ACCCGTCATCTCTGTGAGTG-3′
*Xmag3030*	1	F: 5′-AAAGTGGCGTCCTTCTCTTC-3′
		R: 5′-TGGTGACGATGGCTGACTT-3′
*BE405232*	1	F: 5′-TAGGCAAGTTGCTCTGCTGT-3′
		R: 5′-GATTTTGGGTTGCTCAGCTT-3′
*Xgwm268*	1	F: 5′-AGGGGATATGTTGTCACTCCA-3′
		R: 5′-TTATGTGATTGCGTACGTACCC-3′
*SWES650*	1	F: 5′-GCCGTGCTCCCGTAAACA-3′
		R: 5′-GCTTCACCGACGCAACCT-3′
*TNAC1021-TaqI*	1	F: 5′-CTCATGCATGCGTTTGTTAAA-3′
		R: 5′-CCAGCTGAAACAAGCATCTTC-3′
*BG313767*	1	F: 5′-GAGGCGTTCTTAAGACGGTG-3′
		R: 5′-GGTGTCAAAAACTTCGCCAT-3′
*TNAC1026-HaeIII*	1	F: 5′-GGGATAGAACTCTGGGACTTCA-3′
		R: 5′-AGTGCCAGGGCATAATACAGC-3′
*CINAU971*	2	F: 5′-GAGGGCAGCTTCGACGAC-3′
		R: 5′-CGGCTGGATCACTTCTTCC-3′
*CINAU1616*	7	F: 5′-TGGCCTCAAGTCTCAAGGTT-3′
		R: 5′-ATCCGGCCGTCCAGAAAATA-3′

**Figure 4:**
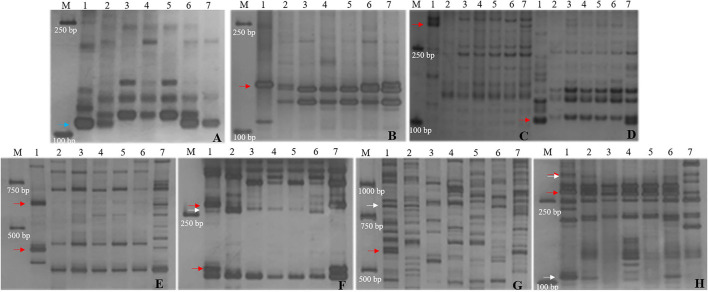
Molecular marker analysis of SN19747 and CH10A5 (specific markers in CH10A5). **(A)** Xcfd63. **(B)** TNAC1044-HaeIII. **(C)** Xwmc432. **(D)** Xwmc93. **(E)** HaeIII-TNAC1088. **(F)** TNAC1021-TaqI. **(G)** BG313767. **(H)** TNAC1026-HaeIII. Lanes: M, DL2000; 1-7 refer to *Th. ponticum*, SNTE20, SNF63, YN15, JM22, SN19647 and CH10A5, respectively. The blue, red and white arrows indicate the bands shared between SN19647 and CH10A5, specific to CH10A5 and SN19647, respectively.

Recently, CH10A5 was reported as a wheat–*Th. ponticum* 1J^S^ (1D) disomic substitution line (Wang et al., 2020). To further compare the 1J^S^ chromosomes in SN19647 and CH10A5, eight markers specific to the 1J^S^ chromosome of CH10A5 were used to screen the two substitution lines. The results showed that only *Xcfd63* produced the same *Th. ponticum* band in SN19647 and CH10A5, while others exhibited differences (Figure 4). Four markers, including two PLUG markers (*TNAC1044-HaeIII* and *TNAC1088- HaeIII*) and two SSR markers (*Xwmc432* and *Xwmc93*), worked only in CH10A5. Two PLUG markers (*TNAC1021-TaqI* and *TNAC1026-HaeIII*) and one EST marker (*BG313767*) showed amplification in both substitution lines but generated different bands specific to *Th. ponticum*.

**Figure 5:**
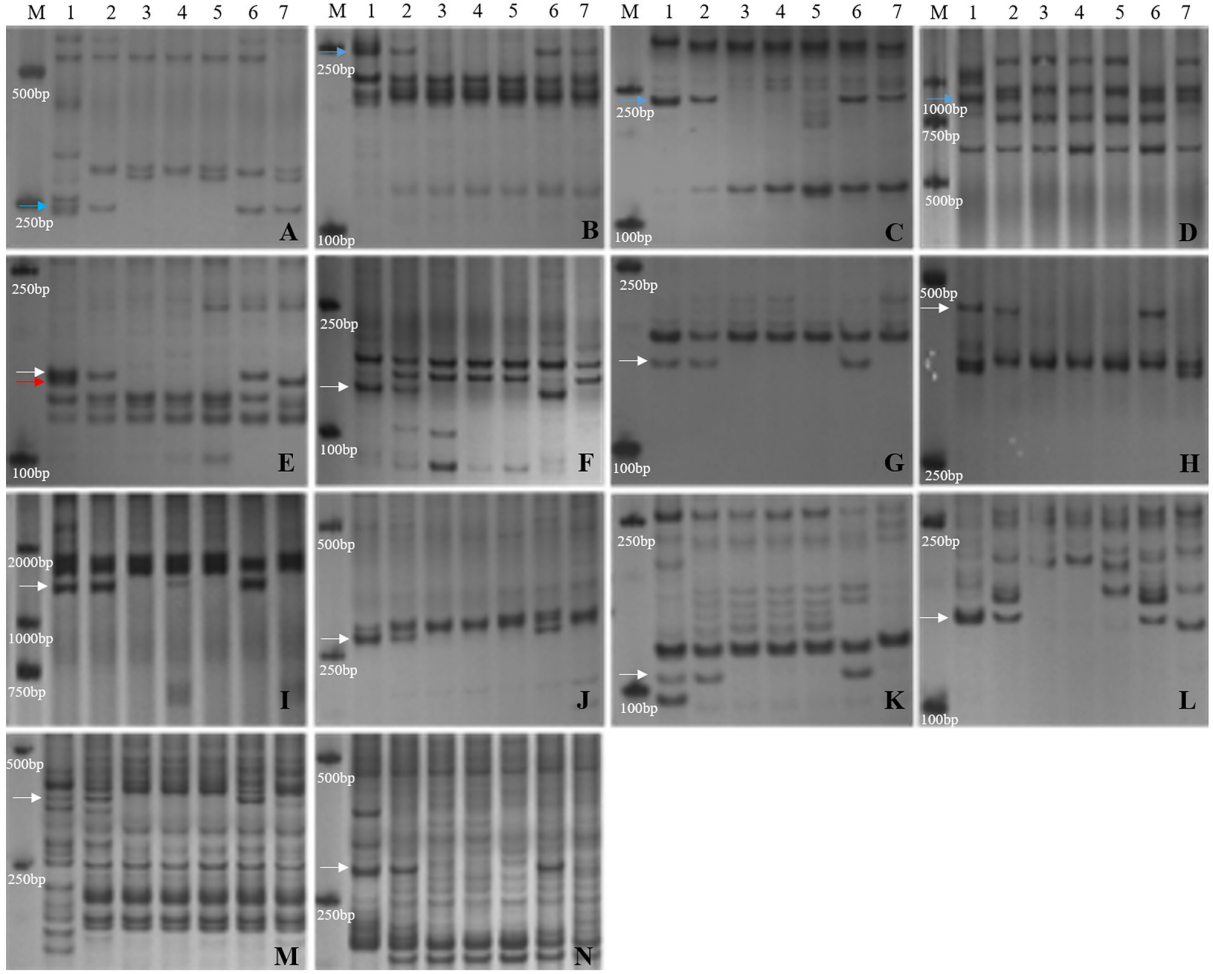
Molecular marker analysis of SN19647 and CH10A5 (specific markers in SN19647). **(A)** CINAU857. **(B)** CINAU889. **(C)** CINAU886. **(D)** CINAU862. **(E)** CINAU880. **(F)** CINAU851. **(G)** CINAU859. **(H)** CINAU865. **(I)** CINAU870. **(J)** CINAU890. **(K)** SWES650. **(L)** Xgwm268. **(M)** BE405232. **(N)** Xmag3030. Lanes: M, DL2000; 1-7 refer to *Th. ponticum*, SNTE20, SNF63, YN15, JM22, SN19647 and CH10A5, respectively. The blue, red, and white arrows indicate the band shared between SN19647 and CH10A5, specific to CH10A5 and SN19647, respectively.

Furthermore, the 14 markers specific to the 1J^S^ chromosome of SN19647 were used to screen the two substitution lines. Four markers produced the same *Th. ponticum* band in SN19647 and CH10A5, and others exhibited differences (Figure 5). Ten markers, including five IT markers (*CINAU851, CINAU859, CINAU865, CINAU870* and *CINAU890*), three EST markers (*SWES650, BE405232* and *Xmag3030*) and one SSR marker (*Xgwm268*), worked only in SN19647. One IT marker (*CINAU880*) worked in both substitution lines but generated different bands specific to *Th. ponticum*. These observations indicated that SN19647 carried a novel pair of 1J^S^ chromosomes different from that of CH10A5. Besides, three new *Th. ponticum-*specific markers were detected in SN19647 (*TNAC1021-TaqI, BG313767*, and *TNAC1026-HaeIII*) compared with CH10A5, among a total of nineteen *Th. ponticum-*specific markers. The results above indicated that the alien chromosomes in SN19647 were different from those in CH10A5.

## Discussion

Gene transfer from wild relatives to common wheat has proven to be an effective approach for improving resistance. As *Th. ponticum* carried resistance to various diseases, many wheats—*Th. ponticum* chromosome lines, including partial amphiploid, addition, substitution and translocation, were developed. Among 11 genes cataloged from *Th. ponticum*, there were 1 and 3 resistant to powdery mildew and leaf rust, respectively. Furthermore, *Pm51* was located on homoeologous 2 (Zhan et al., 2014); *Lr19* and *Lr29* were located on homoeologous 7, and *Lr24* was derived from homoeologous 3 (Li and Wang, 2009). There has been no resistance originating from homoeologous 1 of *Th. ponticum* until the 1J^S^ (1D) substitution line CH10A5 was reported to be resistant to powdery mildew and stripe rust in 2020 (Wang et al., 2020). In this study, the 1J^S^ (1B) substitution line SN19647 exhibited resistance to powdery mildew and leaf rust. This is the first report of leaf rust resistance probably associated with the chromosome 1J^S^ of *Th. ponticum*. As for powdery mildew, SN19647, *Th. ponticum*, SNTE20 and JM22 showed seedling immunity while other parents showed susceptibility. Hence, the origin of powdery mildew resistance is uncertain since JM22 carried *PmJM22* resistant to the isolate E09 at the seedling stage (Yin et al., 2009; Fu et al., 2012). At the adult stage, *PmJM22* seemed to be ineffective, resulting in susceptibility to powdery mildew. Compared to the resistance response with that of the parents, it was deductive that the 1J^S^ chromosomes of *Th. ponticum* in SN19647 was responsible for powdery mildew and leaf rust resistance at the adult stage.

Among 22 markers, involving 8 markers specific to the 1J^S^ chromosomes in CH10A5 and 14 markers specific to the 1J^S^ chromosomes in SN19647, 17 were found to generate different specific bands of *Th. ponticum* between SN19647 and CH10A5. It demonstrated that the 1J^S^ chromosomes in SN19647 were different from those in CH10A5. As known to us, the allodecaploid nature of *Th. ponticum* results in its large and complex genome. Chromosomes belonging to the same genome and homoeologous group may be different ones. According to the study of Chen et al. (1998), *Th. ponticum* possessed 14 pairs of J^S^-genomic chromosomes. Of them, there seemed to be more than one chromosome belonging to homoeologous group one in the J^S^ genome, as shown here.

Fluorescence *in situ* hybridization is a technique that has been widely used to identify chromosome composition and detect specific loci. It has been recently modified to describe and track alien chromosomes introduced into the common wheat background based on the high-resolution karyotype patterns (Du et al., 2017; Kong et al., 2018). In the present study, the FISH pattern of the chromosome 1J^S^ of *Th. ponticum* established 1JS by using the probe combination of AFA-3 (red), AFA-4 (red), pAs1-1 (red), pAs1-3 (red), pAs1-4 (red), pAs1-6 (red), pSc119.2-1 (green), and (GAA)_10_ (green). Additionally, structural variations were detected on wheat chromosomes 2A, 5A, 6B, and 7B. Studies have reported chromosomal recombination, chromosomal modifications, or genomic changes during interspecific hybridization (Liu et al., 2009; Li et al., 2015; Zheng et al., 2015). Moreover, selective breeding leads to structural rearrangements in the chromosomes (Huang et al., 2018). Therefore, the genomic variations detected in SN19647 may have occurred due to the introduction of *Th. ponticum* chromosomes and the subsequent artificial selection for increased adaptability.

Powdery mildew and leaf rust resistance genes transferred from alien genomes of wild Triticeae species are helpful to attenuate the yield losses due to powdery mildew and leaf rust. The development of new resistant materials through distant hybridization will lay the foundation for wheat genetic improvement. Thus, the wheat-*Th. ponticum* 1J^S^ (1B) disomic substitution line SN19647, with resistance to powdery mildew and leaf rust, is a potential resource for exporating positive genes from *Th. ponticum* and breeding disease-resistant wheat lines. In order to improve the usefulness of SN19647, translocations with positive traits are being developed via pollen irradiation.

## Conclusions

A disomic substitution line referred to as SN19647 has a novel pair of 1J^S^ chromosomes from *Th. ponticum*. Its chromosome composition is 14A+12B+14D+2(1J^S^). In the formation process of SN19647, wheat chromosomes 2A, 5A, 6B, and 7B underwent structural variations. Additionally, 19 markers were identified as specific markers to the 1J^S^ chromosome of *Th. ponticum*. Moreover, SN19647 possesses resistance to both powdery mildew and leaf rust. It can serve as positive germplasm for exploring resistance genes from *Th. ponticum* and breeding disease-resistant wheat lines.

## Data Availability Statement

The original contributions presented in the study are included in the article; further inquiries can be directed to the corresponding author.

## Author Contributions

YB designed the research. ML and YW performed the research. XiaL contributed to the disease assessment. XinL and HW contributed to the development of the materials. ML, YW, and YB wrote the manuscript. All authors approved the final version of the manuscript.

## Funding

This work was supported by the National Key Research and Development Program of China (No. 2016YFD0102004-02) and the Major Basic Research Program of Shandong Natural Science Foundation (ZR2019ZD15).

## Conflict of Interest

The authors declare that the research was conducted in the absence of any commercial or financial relationships that could be construed as a potential conflict of interest.

## Publisher's Note

All claims expressed in this article are solely those of the authors and do not necessarily represent those of their affiliated organizations, or those of the publisher, the editors and the reviewers. Any product that may be evaluated in this article, or claim that may be made by its manufacturer, is not guaranteed or endorsed by the publisher.
